# Genotypic and Phenotypic Factors Influencing Drug Response in Mexican Patients With Type 2 Diabetes Mellitus

**DOI:** 10.3389/fphar.2018.00320

**Published:** 2018-04-06

**Authors:** Hector E. Sanchez-Ibarra, Luisa M. Reyes-Cortes, Xian-Li Jiang, Claudia M. Luna-Aguirre, Dionicio Aguirre-Trevino, Ivan A. Morales-Alvarado, Rafael B. Leon-Cachon, Fernando Lavalle-Gonzalez, Faruck Morcos, Hugo A. Barrera-Saldaña

**Affiliations:** ^1^Molecular Genetics Laboratory, Vitagénesis, S.A. de C.V., Monterrey, Mexico; ^2^Evolutionary Information Laboratory, Department of Biological Sciences, University of Texas at Dallas, Richardson, TX, United States; ^3^Departamento de Ciencias Básicas, Centro de Diagnóstico Molecular y Medicina Personalizada, Vicerrectoría de Ciencias de la Salud, Universidad de Monterrey, Monterrey, Mexico; ^4^Servicio de Endocrinología, Hospital Universitario Dr. José E. González, Universidad Autónoma de Nuevo León, Monterrey, Mexico; ^5^Center for Systems Biology, University of Texas at Dallas, Richardson, TX, United States; ^6^Tecnológico de Monterrey, Monterrey, Mexico

**Keywords:** pharmacogenetics, pharmacogenomics, diabetes, sulfonylureas, biguanides, Mexican, direct coupling analysis, direct information

## Abstract

The treatment of Type 2 Diabetes Mellitus (T2DM) consists primarily of oral antidiabetic drugs (OADs) that stimulate insulin secretion, such as sulfonylureas (SUs) and reduce hepatic glucose production (e.g., biguanides), among others. The marked inter-individual differences among T2DM patients’ response to these drugs have become an issue on prescribing and dosing efficiently. In this study, fourteen polymorphisms selected from Genome-wide association studies (GWAS) were screened in 495 T2DM Mexican patients previously treated with OADs to find the relationship between the presence of these polymorphisms and response to the OADs. Then, a novel association screening method, based on global probabilities, was used to globally characterize important relationships between the drug response to OADs and genetic and clinical parameters, including polymorphisms, patient information, and type of treatment. Two polymorphisms, *ABCC8*-Ala1369Ser and *KCNJ11*-Glu23Lys, showed a significant impact on response to SUs. Heterozygous *ABCC8*-Ala1369Ser variant (A/C) carriers exhibited a higher response to SUs compared to homozygous *ABCC8*-Ala1369Ser variant (A/A) carriers (*p*-value = 0.029) and to homozygous wild-type genotypes (C/C) (*p*-value = 0.012). The homozygous *KCNJ11*-Glu23Lys variant (C/C) and wild-type (T/T) genotypes had a lower response to SUs compared to heterozygous (C/T) carriers (*p*-value = 0.039). The screening of OADs response related genetic and clinical factors could help improve the prescribing and dosing of OADs for T2DM patients and thus contribute to the design of personalized treatments.

## Introduction

Type 2 Diabetes Mellitus (T2DM) is the most common form of diabetes in adults. T2DM is associated with multiple complications, such as blindness, lower limb amputation, and premature death (Marchetti et al., 2009; Barquera et al., 2013). According to the International Diabetes Federation (IDF), China, India, United States, Brazil, Russia, and Mexico are the countries with the highest incidence. It is estimated that life expectancy is reduced in diabetic individuals by 5–10 years, mainly due to lack of early treatment. In Mexico, the average age for death by diabetes or its complications was 66.7 in 2010, compared with the lifespan of 76 years of non-diabetic individuals (Agudelo-Botero and Davila-Cervantes, 2015). The average annual economic cost from 2006 to 2010 of T2DM patients in Mexico was $941,345,886 USD of direct cost, $177,220,390 USD of indirect cost, and $27,969,427 USD from its complications. This immense cost, coupled with the issues of inequity and access to healthcare in Mexico, where 51% of the cost comes from household income, represents a huge social burden (Arredondo and De Icaza, 2011; Barquera et al., 2013).

Several classes of oral antidiabetic drugs (OADs) are currently available and primarily include agents that stimulate insulin secretion (sulfonylureas), reduce hepatic glucose production (biguanides), delay the digestion and absorption of intestinal carbohydrate (alpha-glucosidase inhibitors), or improve insulin function (thiazolidinediones) (Krentz and Bailey, 2005; Nathan et al., 2009). Additionally, OADs include other classes of drugs such as meglitinides, glucagon-like peptide-1 (GLP-1) agonists, dipeptidylpeptidase-4 (DPP-4) inhibitors, dopamine-2 agonists, and amylin analogs (Inzucchi et al., 2012). There is a wide variability in adverse events and glucose-lowering response to OADs among different patients, which may be attributed to factors like age, sex, and body weight, but also to genetic variation related to pharmacokinetic and pharmacodynamic properties of the OADs (Becker et al., 2013; Emami-Riedmaier et al., 2015).

Biguanide, especially metformin, which is the only one available OAD in some countries, is recommended as the first-choice therapy for T2DM (Inzucchi et al., 2012). Metformin inhibits the activity of mitochondrial respiratory-chain complex I, resulting in decreased ATP synthesis and an accumulation of AMP leading to the activation of AMP-activated protein kinase (AMPK) and the subsequent suppression of hepatic gluconeogenesis (Foretz et al., 2010). Pharmacokinetic studies suggest that metformin is actively absorbed from the gut and is excreted unchanged in the urine (Zhou et al., 2009). The organic cation transporter 1 (OCT1), encoded by *SLC22A1* gene, is expressed in the basolateral membrane of hepatocytes and mediates the metformin uptake, while OCT2 (encoded by *SCL22A2*), expressed in the basolateral membrane of kidney tubular cells, facilitates almost 80% of metformin excretion (Pearson, 2009; Pernicova and Korbonits, 2014). Associations of intronic variants in *SLC22A1* and *SLC22A2* with glucose-lowering response to metformin in T2DM patients have been previously reported (Tkac et al., 2013). *SLC22A1* gene is highly polymorphic, with common function-reducing polymorphisms such as Arg61Cys (rs12208357), Gly401Ser (rs34130495), and Gly465Arg (rs34059508), which having been associated with decreased transportation and therefore the reduced therapeutic effect of metformin (Distefano and Watanabe, 2010). *In vitro* studies have shown that all three polymorphisms might be associated with reduced metformin uptake (van Dam et al., 2005). However, *in vivo* studies show controversial results (Tzvetkov et al., 2009).

Sulfonylureas (SUs) target an ATP-dependent potassium (K-_ATP_) channel present in pancreatic β-cells. K-_ATP_ channels are hetero-octamers composed of Kir6.2 pore subunit encoded by the gene *KCNJ11*, and the SUR1 receptor subunit encoded by the gene *ABCC8*. SUs lower glycemia by enhancing insulin secretion from pancreatic β-cells by inducing K-_ATP_ channel closure (Tkac, 2015). SUs, such as tolbutamide, glimepiride, and glipizide, are mainly metabolized by the enzyme cytochrome P450 encoded by the *CYP2C9* isoform gene. Several SNPs have been related to their effect on insulin secretion enhancing (Holstein et al., 2005). Reduced drug-metabolizing activity has been reported in individuals carrying two allelic variants namely *CYP2C9*^∗^2 (rs1799853) leading to a missense amino acid polymorphism Arg144Cys, and *CYP2C9*^∗^3 (rs1057910) leading to the missense amino acid polymorphism Ile359Leu (Huang and Florez, 2011). The Ile359Leu polymorphism has a more profound effect (Ragia et al., 2014). These alleles encode proteins with a diminished enzymatic activity and are correlated with elevated serum levels of SUs (Ragia et al., 2009). However, *CYP2C9*-Arg144Cys polymorphism is not associated with diabetes susceptibility (Semiz et al., 2010).

Regarding SUs target (K-_ATP_ channels), most studies researched two linked non-synonymous common variants in both *ABCC8* and *KCNJ11* genes. *KCNJ11* variants are implicated in glycemic progression to either prediabetes or T2DM. One of the most common *KCNJ11* polymorphisms is Glu23Lys (rs5219). The functional effects of the Glu23Lys variant on insulin secretion and sensitivity yield controversial results, even though recent larger studies demonstrate a significantly reduced insulin secretion, lower insulin levels, and improved insulin sensitivity, consistent with the enhanced K-_ATP_ channels activity in pancreatic β-cells (Villareal et al., 2009). More recently, the associations of the Glu23Lys variant and a different *KCNJ11* variant, Ile1337Val (rs5215), with T2DM have been confirmed in several genome-wide association studies (GWAS), rekindling the interest in its potential role as a genetic marker for T2DM development (Cheung et al., 2011). On the other hand, the *ABCC8*-Ala1369Ser (rs757110) polymorphism has been associated with a reduction of glycated hemoglobin (HbA1c) in the Chinese population with SUs treatment (Feng et al., 2008; Sokolova et al., 2015).

In addition to pharmacogenetic factors, the response to OADs is conditional on different phenotypic or clinical aspects. With the accessibility of cohorts of this T2DM patient information, various statistical approaches can be used to determine the contributing factors affecting response to OADs. Traditional statistical tools are used to measure the co-occurrence of factor variable and treatment response at a time (Turner et al., 2009; Stransky et al., 2015). However, the human trait factors may internally relate or function together to affect the drug response. Although these tests provide real statistical connections among variables in patient data, these relationships tend to be composed with both strong and weak correlations making it difficult to disentangle direct effects that explain the influence of some variables over a factor of interest. Therefore, important efforts have been dedicated to the development of statistical models to better describe relationship networks related to human disease. In the field of pharmacogenomics, a variety of statistical models have been built, such as Bayesian networks and Elastic net regression (Barretina et al., 2012), which have exhibited great performance on finding genes highly connected to drug response. Recently, a global statistical model, direct coupling analysis (DCA), also has been demonstrated to be applicable in pharmacogenetic data (Jiang et al., 2017). DCA efficiently computes estimates of a joint probability distribution of multivariate patient profiles constructed with clinical data. The parameters of such distribution estimated by DCA are used to quantify with high success the degree of connectivity of variables in the model. The ability to disentangle direct couplings from indirect couplings has been successful in the field of structural biology where directly coupled residue pairs have been used to predict co-evolution of amino acids (dos Santos et al., 2015), predict the structure of proteins (Sulkowska et al., 2012) with an accuracy not seen before as well as predict the molecular plasticity and complexes (Morcos et al., 2013; dos Santos et al., 2015). Recently, we have used this framework to study protein expression level–based protein–protein interactions and in a pharmacogenomics approach to infer gene–drug interactions in cancer tissues and cell lines where information on drug sensitivity is available (Jiang et al., 2017). This is the first time that direct information (DI) is used as a metric of correlation in high throughput profiling data. It not only captures the connections between well-known drug response predictors, including some drug targets for certain anti-cancer agents, but also predicts some potential biomarkers and generates gene–drug networks. DCA is used in this study to find highly coupled factors for response to OADs and to construct a network for the patient cohort data. A metric called DI is computed to evaluate the association intensity of two variables, including the connections between two potential factors and between factors and drug response.

In addition to genetic variations traits containing pharmacogenetic data, the phenotypic traits of patients, such as age, sex, health status, have been suggested to have influences on the outcome of OADs treatment for T2DM. Thus, a T2DM patient database including genetic data and patient phenotypic data is advantageous. This study collects 495 T2DM patients with information about age, origin, sex, body index, health status, history of OADs treatment, polymorphisms, and results of glycated hemoglobin (HbA1c) tests. HbA1c is a recognized target for diabetes control used in international guidelines and is the most suitable parameter to be studied in pharmacogenetic studies (Lo et al., 2012). Here, we propose a new structure-learning approach for Bayesian network construction by using direct information and Chow-Liu trees. Chow-Liu algorithm is commonly used to learn Bayesian network structure (Almudevar, 2010), and mutual information is used by this algorithm to estimate the dependence of two variables (Chen et al., 2008). Due to the better performance of DI on measuring direct associations when compared to mutual information, we integrated DI and the Chow-Liu algorithm to recover global connections between clinical factors for T2DM patients.

Genetic variations or patient phenotypic data affecting the drug responses to T2DM treatments often lead to the necessity of treatment changes and adjustments, resulting in higher expenses for the patients. The aim of this study was to establish an association between patient clinical data, such as habits, treatment history, polymorphisms, and variability in the response to OAD treatments in a Mexican population. Therefore, biomarkers could help prescribe the right drug and its dosage, for better control of the disease and its consequences, including treatment savings and reduced impact in productivity.

## Materials and Methods

### Design

A cross-sectional and retrospective study with convenience sampling was carried out in T2DM patients treated with OADs, in monotherapy or in combination for at least 6 months, to determine possible association between patient data, gene variants, and drug response assessed by HbA1c values. This study was conducted according to Good Clinical Practice standards and guidelines of the Declarations of Helsinki and Tokyo. Furthermore, the protocol was approved by the Ethics and Research Committee from the Medical School of the Universidad Autonoma de Nuevo León (IRB00005579).

### Patients

We recruited male and female patients with T2DM from northeastern Mexico who attended the Clinic of Diabetes of the Endocrinology Service at the Dr. José Eleuterio González Hospital in Monterrey, Mexico. The recruitment period lasted 12 months. The inclusion criteria were: patients over 18 years old with T2DM and treated with oral antihyperglycemic agents or OADs, in monotherapy or in combination for at least 6 months. The exclusion criteria were: diabetes type 1, gestational diabetes, other non-T2DM types of diabetes, active cancer, heart failure, co-treatment with corticosteroids or estrogens, conditions that can cause hyperglycemia, addiction to alcohol or illegal drugs, and dementia or severe psychiatric disorders. The co-treatments with corticosteroids and estrogens were excluded. The disease status was confirmed using the American Diabetes Association criteria and a physical examination. Blood pressure, body height, and body weight measurement were done. The body mass index (BMI) was calculated from anthropometric measurements.

All patients were apprised about the aims of the study, and a written informed consent was obtained. In addition, information on the history of diabetes and the presence of arterial hypertension, hyperlipidemia, and chronic-degenerative diseases, smoking status, and other medications was obtained from the medical records and from the interview for inclusion in the study.

### Definition of Response

A fasting blood sample was drawn for the determination of HbA1c. HbA1c was measured at least 3 months after drug prescription and determined using Tina-quant^®^ HbA1C Gen. 3 (Cobas-Mira Roche). The approach taken for the treatment of the patients was “treat to target,” defined as failure to reach levels of HbA1c ≤ 7%. The initial HbA1c of each patient was at least 7%.

### DNA Isolation

Peripheral blood from patients was extracted in a tube with EDTA and genomic DNA was isolated with Wizard Genomic DNA Purification Kit (Promega, Madison, WI, United States). Protocol was followed according to manufacturer’s instructions. Genomic DNA was quantified by UV absorbance using Nanodrop (Thermo Scientific, Wilmington, DE, United States). The quality of DNA was measured with the A260/280 ratio, a value of 1.8–2 was considered of good quality. Samples were kept at -20°C in small working aliquots until analysis to avoid recurrent cycles of freezing and thawing to minimize degradation.

### Pharmacogenetic Tests (Genotyping)

A total of 14 single nucleotide polymorphisms distributed in 5 different genes associated with response to anti-diabetic treatments were genotyped by Real-Time PCR system using validated Genotyping Assays (Applied Biosystems, Foster City, CA, United States) according to the manufacturer’s instructions. Two additional polymorphisms in *SLC22A1* gene (Met61Val and Met420Del) were included in the study and analyzed in 50 responders and 50 non-responder patients. These additional polymorphisms were determined by nucleotide sequencing method in a Genetic Analyzer 3100 (Applied Biosystems). As a quality control measure, genotyping for the polymorphisms were required to pass three tests for inclusion in subsequent association studies: the genotype call rate (> 0.90 completeness to obtain 99.8% accuracy), the Hardy-Weinberg equilibrium (HWE) test (*p*-value > 0.05), and the minor allele frequency (MAF) criterion (> 0.01).

### Analysis of Statistical Significance

Standard descriptive and comparative analyses were performed. The responder’s phenotypes classification was made using Hb1Ac parameter applied a cut-off ≤ 7 for responder’s and > 7 for non-responder’s [including first-line therapy (FLT), second-line therapy (SLT), third-line therapy (TLT), monotherapy, and combination therapy]. The HWE was determined by comparing the genotype frequencies with the expected values using the maximum likelihood method. To detect significant differences between two groups, Student’s *t*-test or the Mann–Whitney *U*-test were used for parametric or non-parametric distributions, respectively. Differences between more than two groups were assessed by one-way ANOVA and the Kruskal–Wallis *H*-test for parametric or non-parametric distributions, respectively. *Post hoc* tests (LSD and Tamhane’s T2) were used for pairwise comparisons. Possible associations between genotypes and phenotypes were assessed using contingency tables *X*^2^ statistics and Fisher’s exact tests. The association was evaluated under four different models (dominant, over dominant, recessive, and additive). Odds ratios were estimated with 95% confidence intervals. Aforementioned analyses were performed with SPSS for Windows, V.20 (IBM Corp., Armonk, NY, United States). All *p*-values were two-tailed. The corrected *P* (*P*c)-values were adjusted by using Bonferroni’s correction. A *p*-value ≤ 0.05 was considered statistically significant.

### Computational Modeling: Direct Coupling Analysis

To study the association between diabetes-related SNPs, patient data and antidiabetic drug response, we have developed a metric called DI, which is derived from the inference framework DCA (Morcos et al., 2011). DCA is a statistical method that infers efficiently the parameters of probability distributions with a large set of variables. DCA can be computed efficiently and is able to capture and evaluate direct pairwise correlations among potentially thousands of variable connections. The probability distribution of large sets of data is modeled with the following Boltzmann-like distribution:

$$\begin{array}{c} P(dat)=\frac{1}{Z}\exp\{\sum e_{ij}+\sum h_{i}\} \end{array}$$

where *dat* represents a profile with *L* variables that are indexed by *i* and *j* and *Z* is a normalization constant. The parameters of this distribution are all possible *e_ij_* and *h_i_* for *i, j* ≤*L* and contain information about pairwise direct connectivity (*e_ij_*) of the variables in the dataset. They are typically hard to be calculated exactly, but can be estimated using DCA. Once the parameters have been estimated, we can use them to compute pairwise probabilities. The following expression shows the form of DI based on the probabilities computed using the parameters, *e_ij_* and *h_i_.*

$$\begin{array}{c} DI_{ij}=\sum_{x_{i},x_{j}}P_{ij}(x_{i},x_{j})\log\frac{P_{ij}(x_{i},x_{j})}{f_{i}(x_{i})f_{j}(x_{j})} \end{array}$$

Here *x_i_* is the quantized value of the clinical variable in the profile. The values of the *DI_ij_* pairs tell us how connected are two variables in the distribution.

### Analysis on T2DM Patient Data

**FIGURE 1:**
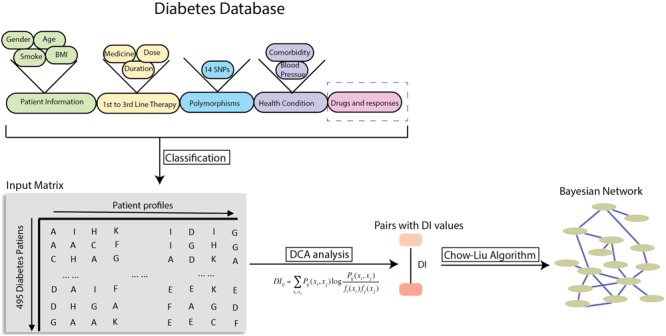
Workflow of global probabilistic modeling on T2DM patient data. Strategy of using T2DM patient datasets to compute the direct information metric between patient genetic or clinical factors and the drug response of OAD treatments. After DI values were calculated, they were used in the Chow-Liu tree for a structural learning for Bayesian network.

The DCA was applied to the complete cohort of data as described in **Figure 1**. The responder’s phenotypes classification was made at a cut-off 7 as defined before. Patient’s body indexes, such as weight, height, BMI, age, duration of diabetes, systolic pressure, diastolic pressure, are classified based on decade spans. To find the influential factors for response to OADs, a matrix containing all patient phenotypic informatics, 14 polymorphisms, HbA1c test result is generated as the input for DCA algorithm (Morcos et al., 2011). The T2DM database consists of patient profiles from 495 patients, including basic information, first, second, third line therapy information, 14 polymorphisms, health conditions, and the HbA1c test result estimating the glucose-lowering effect of OADs. The patient profile columns also include the 21 OADs separately, representing the usage and doses of a specific OAD for certain patients. All of those profiles data are classified and organized in an input matrix for DCA. DI is computed from DCA as a metric of connectivity strength for pairwise variables. The higher DI values, the stronger the correlation between these two variables. DI has been successfully applied to model molecular interactions in protein folds (Morcos et al., 2011,Morcos et al., 2014; dos Santos et al., 2015; Boyd et al., 2016) as well as to identify drug-gene connections in cancer datasets (Jiang et al., 2017). Then, DI values for each variable pair is computed by DCA algorithm and then is used to find a complete network by using a minimum spanning tree approach and then a Bayesian network is built with undirected edges.

### Predictive Model for OAD Treatment Response

The direct connectivity (*e_ij_*) estimates the strength of couplings between two variables at certain states. The summation of *e_ij_* over all of patient profile factors with drug response provides a score to evaluate each patient’s glucose lowering response after taking OADs under his specific genetic and clinical profiles.

When summing all the *e_ij_* with the *j* defined as the HbA1c level ≤ 7%, the Score represents how likely the patient is responding to the current OAD treatment based on his/her body indexes, treatment strategy, polymorphisms, health condition.

$$\begin{array}{c} Score_{Res}=\sum_{i}e_{ij}(x_{i},Res) \end{array}$$

where *i* denotes a genetic or clinical factor of patient, and *x_i_* represents the class of the factor belongs to. Additionally, the score for a patient’s inert responses to the OAD is calculated based on the *e_ij_* with *j* representing HbA1c level > 7%.

$$\begin{array}{c} Score_{NonRes}=\sum_{i}e_{ij}(x_{i},NonRes) \end{array}$$

The two scores for each patient are compared and the treatment response is predicted based on which score is larger. The leave one out cross-validation is conducted to evaluate the performance of this predictive model.

## Results

### Descriptive Statistics and Phenotype Classification

A total of 495 patients treated with hypoglycemic drugs were included in this study. The subjects were Mexican, mainly from northeastern of Mexico. The average age of patients was 56.30 ± 12.16 for males and 56.41 ± 11.45 for females. No significant differences were found for the age of diagnosis, diabetes duration, and HbA1c values between males and females. However, the BMI was statistically higher in females (**Table 1**). Regarding to co-morbidities, the most frequent co-morbidity was hypertension with 24.4%, followed by hypertension-dyslipidemia with 13.1%, only dyslipidemia (7.5%), hypothyroidism (6.3%), and hypertension-hypothyroidism (4.6%).

**Table 1:** Demographic and clinical data of patients.

Patients	*N*	Age	Diagnosis age	Diabetes duration	BMI	HbA1c
Males	156 (31.5%)	56.30 ± 12.16	45.12 ± 12.013	11.45 ± 8.03	28.90 ± 4.46£	8.69 ± 2.24
Females	339 (68.5%)	56.41 ± 11.45	45.32 ± 10.705	10.95 ± 8.63	30.66 ± 6.78	8.45 ± 2.10
Non-responders	353 (71.3%)	56.30 ± 11.51	44.14 ± 11.00∆, ¥	12.14 ± 8.25 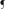	29.83 ± 5.95	9.40 ± 1.92£,§
MT non-responders	332 (67.1%)	56.47 ± 11.48	44.39 ± 10.95	11.00 ± 8.26	30.00 ± 6.07	9.33 ± 1.94
CT non-responders	30 (6.1%)	55.27 ± 11.51	42.53 ± 11.23	13.07 ± 8.28	28.50 ± 4.80	9.6 ± 1.81
Responders (any type)	142 (28.7%)	56.56 ± 12.09	48.02 ± 10.98	8.54 ± 8.39	30.79 ± 6.72	6.34 ± 0.47
MT responders	127 (25.7%)	55.88 ± 12.08	47.92 ± 11.36	8.06 ± 7.88	30.91 ± 6.72	6.32 ± 0.43
CT responders	7 (1.4%)	65.29 ± 13.16	49.29 ± 6.90	15.43 ± 14.26	25.83 ± 3.78	5.97 ± 0.79
FLT responders	93 (18.8%)	56.61 ± 11.11	49.68 ± 10.83*i*	6.92 ± 6.89Ȼ	31.46 ± 6.85	6.30 ± 0.50
SLT responders	39 (7.9%)	56.77 ± 12.98	46.62 ± 10.33	10.19 ± 8.79	30.13 ± 6.15	6.43 ± 0.41
TLT Responders	10 (2.0%)	55.20 ± 17.69	38.10 ± 9.67	17.10 ± 13.07	27.13 ± 6.88	6.40 ± 0.44

*Data presented as mean ± SD. BMI: body mass index; HbA1c: hemoglobin A_*1c*_; MT: monotherapy; CT: combined therapy; FLT: first-line therapy; SLT: second-line therapy; TLT: third-line therapy. ^ϵ^*p* = 0.025 (male vs. female), ^Δ^*P* = 4.25 × 10^-*4*^ (non-responders vs. responders), ^
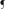
^*p* = 2.5 × 10^-*7*^ (non-responders vs. responders), ^£^*p* = 6.29 × 10^-*68*^ (non-responders vs. responders), ^¥^*p* = 1.41 × 10^-*4*^ (non-responders vs. FLT), ^Ϸ^*P* = 0.025 (FLT vs. TLT), Ȼ*p ≤* 0.049 (FLT vs. non-responders, SLT, and TLT), and ^§^*p ≤* 6.84 × 10^-*8*^ (non-responders vs. FLT, SLT, TLT).*

The phenotype classification based on HbA1c values (**Table 1**) was significantly different between the responder’s and non-responder’s (*p* = 6.29 × 10^-68^). More than half of the patients (353) did not respond to any type of therapy (HbA1c > 7%), failing in 71.3% of the cases, and the treatment was effective (HbA1c ≤ 7%) in 142 individuals. The average diagnosis age of non-responders showed significant lower values (*p* = 4.25 × 10^-4^) compared to responder’s, but showed statistically significant higher values of diabetes duration (*p* = 2.5 × 10^-7^). A total of 93 patients (18.8%) responded to FLT, and they showed higher values of diagnosis age (*p* = 0.025), although for lower values of diabetes duration (*p* ≤ 0.049), compared to responder’s to TLT. None other therapies had a significant difference.

The drug most commonly used for the FLT was metformin in monotherapy (46.7%). The second most used drug in FLT was a SU in combination with metformin (34.6%). For SLT and TLT, metformin was also very commonly used (16.7 and 8.0%, respectively). For FLT, SLT, and TLT, the third most common option was SU in monotherapy (9.3, 13.3, and 5.3%, respectively). Insulin was the most common treatment choice in SLT and TLT (55.2 and 69.3%, respectively), although it was the fifth option in FLT (2.2%) (**Table 2**).

**Table 2:** Scheme for the treatment of T2DM.

	First-line therapy		Second-line therapy		Third-line therapy	
Drug	*N*	Percent	*N*	Percent	*N*	Percent
Metformin	231	46.7	45	16.7	6	8
Metformin/Sulfonylurea	171	34.6	3	1.1	1	1.3
Sulfonylurea	46	9.3	40	14.8	5	6.8
Other	36	7.2	33	12.2	11	14.6
Insulin	11	2.2	149	55.2	52	69.3
Total	495	100	270	100	75	100

### Pharmacogenetic Findings by Standard Statistical Methods

The polymorphisms M165I and R400C in *SLC22A2* gene were not in HWE equilibrium. The SNPs G401S and R465G in *SLC22A1* gene, and K432Q in *SLC22A2* gene, had a Minor Allele Frequency (MAF) < 0.01. The polymorphisms were excluded from subsequent analyses. As a result a total of 9 SNPs remained for statistical analysis. Two polymorphisms, Ala1369Ser in gene *ABCC8* and Glu23Lys in gene *KCNJ11*, showed a significant impact on response to SUs.

The effect of *ABCC8*-Ala1369Ser polymorphism on Hb1Ac under SU treatment was statistically significant. Heterozygous variant (C/T) carriers had lower HbA1c values compared to homozygous wild-type (A/A) carriers (*p* = 0.029) and compared to homozygous wild-type and variant (A/A+C/C) carriers (*p* = 0.012). The genotypes resulting from the *KCNJ11*-Glu23Lys polymorphism also had a significant impact on HbA1c under SU treatment. First, the homozygous wild-type and variant (C/C+T/T) carriers had higher HbA1c values (*p* = 0.039) as compared to heterozygous carrier (C/T). None of the other 7 polymorphisms tested had a significant impact on clinical parameters (**Table 3**).

**Table 3:** Association values between gene polymorphisms and clinical parameters.

Polymorphism	*N*	BMI	Diagnosis age	HbA1c
***ABCC8*-Ala1369Ser**				
A/A	180	30.74 ± 6.84	46.23 ± 11.37	8.69 ± 2.07^Δ^
A/C	241	29.84 ± 5.93	45.16 ± 11.34	8.34 ± 2.21
C/C	74	29.44 ± 5.26	43.20 ± 9.52	8.74 ± 2.09
A/A+C/C	254	30.36 ± 6.44	45.35 ± 10.93	8.70 ± 2.07^ 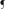 ^
***CYP2C9*-Arg144Cys**				
C/C	423	30.07 ± 6.26	45.41 ± 11.03	8.49 ± 2.17
C/T	67	30.30 ± 6.04	44.69 ± 11.40	8.67 ± 2.00
T/T	5	30.17 ± 2.09	40.00 ± 16.33	9.60 ± 2.13
***CYP2C9*-Ile359Leu**				
A/A	460	30.06 ± 6.01	45.35 ± 10.96	8.52 ± 2.13
C/A	35	30.67 ± 8.28	44.06 ± 13.24	8.55 ± 2.34
***KCNJ11*-Glu23Lys**				
C/C	179	30.70 ± 6.87	46.26 ± 11.56	8.64 ± 2.08
C/T	246	29.91 ± 5.96	44.95 ± 11.25	8.37 ± 2.19
T/T	70	29.26 ± 5.01	43.79 ± 9.28	8.75 ± 2.13
C/C+T/T	249	30.29 ± 6.42	45.56 ± 11.00	8.67 ± 2.09^£^
***KCNJ11*-Ile1337Val**				
C/C	71	29.36 ± 5.05	43.75 ± 9.22	8.74 ± 2.12
C/T	247	29.97 ± 6.17	44.91 ± 11.34	8.38 ± 2.20
T/T	177	30.59 ± 6.62	46.34 ± 11.46	8.64 ± 2.07
***SLC22A1*-Arg61Cys**				
C/C	475	30.05 ± 6.21	45.40 ± 11.03	8.53 ± 2.16
C/T	20	31.43 ± 5.83	41.90 ± 13.02	8.51 ± 1.77
***SLC22A1*-Met61Val**				
G/G	92	30.77 ± 6.22	46.41 ± 9.96	8.18 ± 2.04
A/G	26	29.74 ± 4.45	45.58 ± 14.77	8.14 ± 1.74
A/A	6	28.66 ± 4.03	43.67 ± 10.65	8.15 ± 1.83
***SLC22A2*-Ala270Ser**				
A/C	58	30.04 ± 6.35	46.03 ± 11.17	8.04 ± 1.56
C/C	437	30.11 ± 6.18	45.15 ± 11.12	8.59 ± 2.20
***SLC22A2*-Met420Del**				
ATG/ATG	52	30.56 ± 6.30	44.65 ± 9.87	8.35 ± 2.09
ATG/delTGA	49	30.72 ± 5.77	48.10 ± 12.73	8.05 ± 2.00
delGAT/delGAT	23	29.61 ± 4.78	45.13 ± 9.59	8.02 ± 1.56

*Data presented as mean ± SD. BMI: body mass index; HbA1c: hemoglobin A1c. ^Δ^*P* = 0.029 (A/A vs. A/C), ^
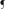
^*p* = 0.012 (A/A+C/C vs. A/C), and ^£^*p* = 0.039 (C/C+T/T vs. C/T).*

The association was evaluated under genetic models for only nine polymorphisms that had passed a quality control. We found that two of the nine polymorphisms were associated with the responder phenotype. The A/C genotype of *ABCC8*-Ala1369Ser and the C/T genotype of *KCNJ11*-Glu23Lys were significantly associated with responder phenotype using over dominant model. This association remained statistically significant after adjusting using Bonferroni’s correction (*p* < 0.05) (**Table 4**).

**Table 4:** Association values between genotypes and response using dominant, over-dominant, and additive models.

Gene	Polymorphism	Model	OR (95% CI)	*p*-value	*P*c-value
*ABCC8*	Ala1369Ser	Over-dominant (A/A+C/C vs. A/C)	A/A+C/C = 1.33 (1.11–1.59)	0.03	0.04^∗^
			A/C = 0.736 (0.59–0.92)		
*KCNJ11*	Glu23Lys	Over-dominant (C/C+T/T vs. C/T)	C/C+T/T = 1.27 (1.06–1.51)	0.013	0.018^∗^
			C/T = 0.77 (0.62–0.96)		

*OR: odds ratio; CI: confidence interval; *P*c: *P*-values adjusted by using Bonferroni’s correction for multiple comparisons; ^∗^*p* ≤ 0.05.*

### Pharmacogenetic and Clinical Parametric Findings From T2DM Patient Profiles by Direct Coupling Analysis

The DCA finds factor-drug response connections from a global statistical model computed from an estimate of the joint probability distribution of all clinical variables in the study. **Figure 1** shows the classification process that the patient clinical and genetic data undergoes to form the input discrete matrix for DCA algorithm. The outcome is a set of pairs with DI values. To uncover the minimal set of relevant connections between those factors, a Bayesian network is constructed by using the Chow-Liu algorithm as shown in **Figure 1**. However, this study refines Chow-Liu algorithm by replacing the typical use of mutual information with DI from DCA to calculate the Kullback–Leibler distance. This is a novel approach to generate the Bayesian network. Some factors cluster together and are connected showing previously known relationships, such as the connections between weight, height, BMI, and gender. These known associations of factors can be seen as validation of the links found by the algorithm. The time lengths of treatment (first line and second line), age, age of diagnosis, and diabetes diagnosis span are clustered; however, the treatment history for the third line therapy is more likely to be associated with weight.

**FIGURE 2:**
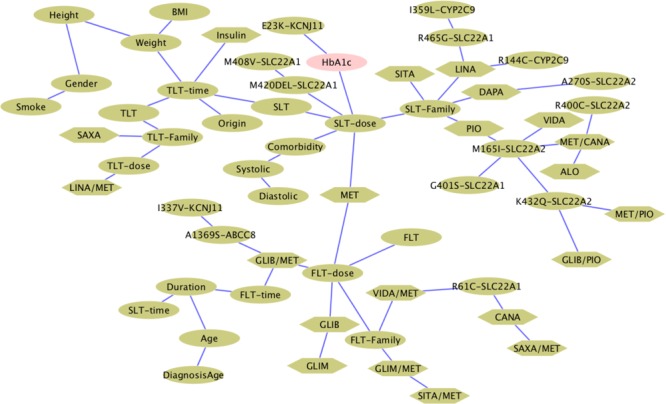
Bayesian network of OADs and factors built from direct information. Hexagonal shapes indicate OADs and ovals denote clinical parameters or polymorphisms.

In agreement with the pharmacogenomics finding that *KCNJ11* Glu23Lys affects the response to SUs, while *KCNJ11* Glu23Lys is generally connected to response to OADs. However, the *ABCC8* Ala1369Ser variant is not connected to any drug in this network and is linked to *KCNJ11* Ile1337Val variant. Polymorphisms in the *SLC22A2* gene have been identified and shown to cause inter-patient variability in the pharmacokinetic and pharmacodynamic profile of metformin. Three gene variants, M165I (rs8177507), Ala270Ser (rs316019), and R400C (rs8177516), of the *SLC22A2* gene were reported with reduced uptake of OCT2 substrate, whereas a fourth one, K432Q (rs8177517), showed an increased uptake activity compared to the wild-type allele. However, attempts to translate those findings into altered response to metformin of diabetic patients in several populations have not been successful (Meyer zu Schwabedissen et al., 2010). As shown in **Figure 2**, 3 out of 4 polymorphisms in *SLC22A2* have connections to metformin in combination with other drugs. The genetic variants of *SLC22A2* identified in a Korean population appear to have a significant impact on the disposition of metformin. As expected from the primary distribution of OCT2 in the kidney, the tubular excretion was influenced mainly by the M165I, Ala270Ser, and R400C variants of *SLC22A2*, leading to an increase in plasma metformin concentrations in subjects with these variants (Song et al., 2008). MET is connected to FLT cluster and SLT cluster, being consistent with the fact that MET is the most commonly used drug in FLT and the second common drug in SLT. Two SU drugs, GLIB and GLIM, are connected together.

**FIGURE 3:**
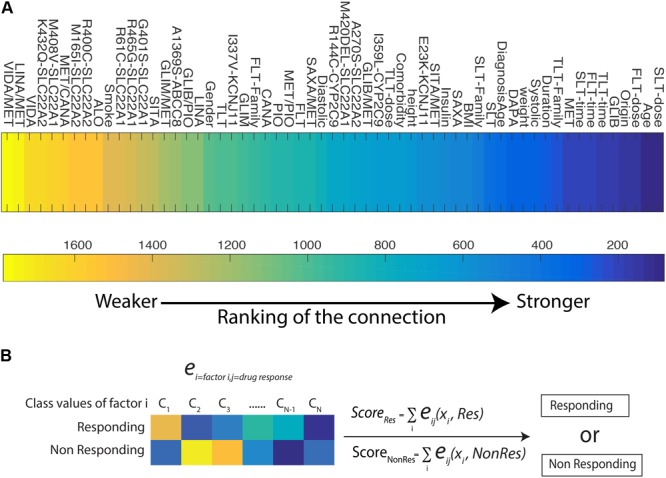
**(A)** Heat map for the ranking of each factors and HbA1c test result among all the pairwise pool. The lower value of the ranking indicates stronger connectivity. **(B)** A predictive model for patient’s drug response to OAD treatments.

To systematically investigate the connection between blood glucose lowering outcome and other factors, we studied the couplings between those factors and the drug response HbA1c test results. In the input matrix, the values in columns for each drug identify their presence or absence in the treatment. The overall ranking of each drug response connection is shown in the heatmap of **Figure 3A**. Treatment time and doses are highly associated with HbA1c results. Age and place of origin appear to be strongly influential. The administration of GLIB or MET in monotherapy is also highly connected to HbA1c results, partially corresponding with the fact that Metformin is the most commonly used treatment for T2DM. Among the body indexes parameter, weight and BMI still have high rankings, which suggests that in prediction of treatment outcome those two factors are worthy of consideration. The rankings of polymorphisms have the highest influence at *KCNJ11* Glu23Lys, which is observed to be correlated with drug response to SUs in both the statistical significance study and the DI-based Bayesian network.

In order to predict the glucose-lowering efficacy of each OADs and determine a better therapy strategy based on a given profile of patient, we develop a predictive model on DI (**Figure 3B**). DI is a metric of direct coupling among variables but it does not reveal the directionality of this connection. It is possible to use the parameters of the global joint distribution, to quantify how a large number of factors account for a possible outcome, i.e., responsive or non-responsive treatment. This additive model uses the *e_ij_(x_i_,x_j_)* estimates connecting factors to response with the aims to distinguish between the responder and non-responder group. We conducted a leave one out cross-validation on the 495 T2DM patients dataset, and reached an average of prediction rate at 0.70, with the maximum response vs. non-response prediction rate at 0.76.

## Discussion

### Association Between Gene Polymorphisms and Clinical Parameters

From the nine analyzed pharamcogentic polymorphisms seeking to explain the relationship between diverse genotypes of diabetic patients and their response to different OADs, only two polymorphisms, *ABCC8*-Ala1369Ser and *KCNJ11*-Glu23Lys, showed a significant impact on response on the reduction of Hb1Ac with SU treatment. None of the other seven polymorphisms tested had a significant impact on clinical parameters. These results confirm the association of *ABCC8*-Ala1369Ser polymorphism and reduction of HbA1c level in the Chinese population with SU treatment (Feng et al., 2008). Nevertheless, studies in Caucasian populations showed no association of *KCNJ11*-Glu23Lys with Hb1Ac reduction in response to SUs (Ragia et al., 2012).

The *CYP2C9* polymorphisms included in this study, Arg144Cys and I1359L, showed no significant differences in response to SUs in comparison with studies carried on Caucasian population in which they described a higher sensitivity to SUs for Ile359Leu and Arg144Cys variant carriers (Becker et al., 2008; Ragia et al., 2014). The *KCNJ11*-I337 polymorphism showed no evidence of being related in the response to SUs as a study carried on Chinese population suggests (Cheung et al., 2011). The *SLC22A1* polymorphisms, Arg61Cys and Met61Val, showed no significant evidence of being related in the response to metformin in comparison with a study carried in Caucasian population in which they found a significant reduction of Hb1Ac after 6 months of metformin treatment (Tkac et al., 2013). The *SLC22A2* polymorphisms showed no evidence of being related in the response to metformin, contrary of what has been suggested (Avery et al., 2009).

### Association Between Genotypes and Phenotypes

Only the A/C nucleotide change from polymorphism Ala1369Ser (gene *ABCC8*) and the C/T nucleotide change from polymorphism Glu23Lys (gene *KCNJ1*) were significantly associated with responder phenotype using an over dominant model. *KCNJ11* and *ABCC8* encode for the subunits KIR6.2 and SUR1, respectively, of the heteroctomer K_ATP_ channel (Emami-Riedmaier et al., 2015). K_ATP_ channels regulate membrane K^+^ flux for various cell types including pancreatic β-cells, where increased glucose metabolism results in the closure of the K_ATP_ channels leading to calcium influx and subsequent insulin secretion (Nathan et al., 2009). Notably, *KCNJ11* and *ABCC8* genes lie close to each other on chromosome 11, with strong linkage disequilibrium. In a Caucasian population study, Ala1369Ser was correlated with Glu23Lys, where for every K allele of *KCNJ11* gene found there was A allele of *ABCC8*, thus constituting a possible haplotype (Florez et al., 2004), whereas several studies and meta-analyses showed the association of *KCNJ11*, but not of *ABCC8* polymorphisms, with susceptibility to type 2 diabetes (van Dam et al., 2005; Gong et al., 2012).

We showed that it is possible to use patient data in this comprehensive study to generate a model of the global distribution of patient profiles. This model includes phenotypic factors, health conditions, treatment information, and polymorphisms with clinical treatment outcome variable. Although we found agreement between the standard statistical tests and the global pairwise DCA model about how *KCNJ11*-Glu23Lys affects the efficacy of SUs drug, we also found novel relationships when modeling the dataset with global techniques. We uncover a network connecting OADs, gene polymorphisms, and patient information. Connections with the HbA1c test and metrics for the association between each pairwise variables can inform better how a large set of factors interact during disease progression.

A predictive model for OAD drug response is proposed based on direct coupling parameters *e_ij_* in this study and its predictive performance has been validated by cross validation. The overall prediction rate both for predicting as responding or non-responding can be as high as 0.76. This model has the potential to be used as a guide to modify factors to predict higher response scores. This is a topic of further research that can have applications in personalized therapies. With increasing well-phenotyped cohorts and new methods, such as Next Generation Sequencing and global statistical analyses, the next few years promise a renewed interest in the use of pharmacogenetics to unravel drug and disease mechanisms, as well as the possibility to individualize T2DM therapy by genotype.

## Author Contributions

HB-S and LR-C conceived of the idea of analyzing genetic factors altering the oral antidiabetic drugs response in Mexican type II diabetes patients. LR-C, CL-A, HS-I, and IM-A carried out the real-time PCR experiments and Sanger sequencing experiments. FL-G provided the biospecimens and their clinical data. FM, X-LJ, and RL-C analyzed the data and designed the models and the computational framework. X-LJ and FM developed the predictive model. HB-S was in charge of overall direction and planning. HS-I, FM, X-LJ, LR-C, and DA-T wrote the manuscript. HB-S and FM should be considered as co-corresponding authors.

## Conflict of Interest Statement

The authors declare that the research was conducted in the absence of any commercial or financial relationships that could be construed as a potential conflict of interest.
